# Safety of live attenuated varicella-zoster vaccine in patients with underlying illnesses compared with healthy adults: a prospective cohort study

**DOI:** 10.1186/s12879-019-3719-7

**Published:** 2019-01-28

**Authors:** Satoko Ohfuji, Kazuya Ito, Megumi Inoue, Motoki Ishibashi, Hiroko Kumashiro, Yoshio Hirota, Eiji Kayano, Naoshi Ota

**Affiliations:** 1 Department of Public Health, Osaka City University Graduate School of Medicine, 1-4-3, Asahi-machi, Abeno-ku, Osaka-city, Osaka 545-8585 Japan; 2 Research Center for Infectious Disease Sciences, Osaka City University Graduate School of Medicine, 1-4-3, Asahi-machi, Abeno-ku, Osaka-city, Osaka 545-8585 Japan; 3 PS Clinic, Medical Co. LTA, 6-18, Ten-ya-machi, Hakata-ku, Fukuoka-city, Fukuoka 812-0025 Japan; 4 Clinical Epidemiology Research Center, Medical Co. LTA, 3-6-1 Kashii-Teriha Higashi-ku, Fukuoka, 813-0017 Japan; 5 Kanonji Institute, Research Foundation for Microbial Diseases of Osaka University, 4-1-70, Seto-Cho, Kanonji, Kagawa 768-0065 Japan

**Keywords:** Adverse events, Autoimmune diseases, Diabetes mellitus, Herpes zoster, Malignancy, Reactogenicity, Renal diseases, Safety, Varicella-zoster vaccine

## Abstract

**Background:**

In Japan, freeze-dried live attenuated varicella-zoster vaccine is available for adults aged ≥50 years to prevent herpes zoster. However, limited evidence has been accumulated regarding vaccine safety for patients with underlying illnesses, who have been considered as the high-risk group for herpes zoster.

**Methods:**

A prospective cohort study of 1200 healthy adults and 300 patients with underlying illnesses such as malignancy, diabetes mellitus, autoimmune diseases, and renal diseases was conducted. All subjects were vaccinated and then their adverse events (AEs) were followed for 28 days after vaccination. Key safety measures included any AEs, severe AEs (SAEs), and vaccine-related AEs such as injection-site AEs and systemic AEs. The frequencies and 95% confidence intervals of AEs were calculated.

**Results:**

During the follow-up period, 2 SAEs (bone fracture and acute cholecystitis) among healthy adults and 1 SAE (disseminated mycobacteriosis) among patients with underlying illnesses were reported, although none of them was diagnosed as vaccine-related. Vaccine-related AEs were reported in 42% of healthy adults and patients with underlying illnesses, and the proportions were similar between the groups. The most frequent AEs were injection-site AEs in both groups (i.e., 41 and 39%), and systemic AEs were observed in 4% of both groups. Only among healthy adults, those with a history of herpes zoster were more likely to report injection-site AEs than those without a history of herpes zoster (53% vs 39%).

**Conclusions:**

The present study confirmed the safety of freeze-dried, live attenuated varicella-zoster vaccine even in patients with underlying illnesses. A history of herpes zoster might be related to development of injection-site AEs in healthy adults.

**Trial registration:**

The study was prospectively registered on Japic-Clinical Trials Information as JapicCTI-163415 on October 31, 2016.

## Background

Herpes zoster (HZ), or shingles, is one of the important diseases that could decrease quality of life of older adults. It is caused by reactivation of varicella-zoster virus (VZV) in individuals with latent infections, and is characterized by unilateral radicular pain and a vesicular rash generally limited to a single dermatome, corresponding to the sensory ganglion in which the latent VZV reactivated [1]. It can expand to involve several dermatomes, especially in immunocompromised subjects. The frequency and severity of HZ increase with age, which correlates closely with a progressive decline in cell-mediated immunity to VZV [2]. The most common complication is post-herpetic neuralgia (PHN), which is a very problematic condition because it is often difficult to control the intolerable pain and results in decreased quality of life for the affected individuals.

The incidence of HZ has been increasing with the current ageing population in Japan. According to a Japanese study using medical records between 1997 and 2006, the annual incidence of HZ was 1.96–2.85/1000 person-years among individuals below the age of 50 years, but it increased to 5.23–7.84/1000 person-years among those aged 50 years or more [3]. The more recent studies in Japan indicated that the incidence of HZ among older individuals has been increasing to 10.2/1000 person-years [4] or 10.9/1000 person-years [5].

Patients with diabetes mellitus, autoimmune diseases, renal failure, and malignancies have a higher risk of HZ than those with other diseases [6, 7]. In addition, the proportion developing PHN among HZ patients ranged from 9% [4] to 19% [5], and its risk was increased in males, age ≥ 65 years, and immunosuppressive therapy [4]. Thus, it is important to protect these high-risk populations from the threat of HZ and PHN.

Since immunity to VZV plays a role in the pathogenesis of HZ [1], ZOSTAVAX® (Merck & Co., Inc) as a live attenuated virus vaccine for Oka strain (19,400 PFU or more, based on the package insert) has been approved in more than 60 countries or counties for prophylactic use in older individuals. The clinical efficacy was reported to be 51.3% for reducing the incidence of HZ and 66.5% for reducing the incidence of PHN [8]. In Japan, freeze-dried live attenuated varicella vaccine for Oka strain (1000 PFU or more, based on the package insert), which was originally used to prevent varicella in children since 1986, was additionally approved for use to reduce the risk of HZ in individuals aged ≥50 years in 2016. Since this varicella-zoster vaccine generally contains live attenuated Oka virus of 23,000–95,000 PFU [9], the identical vaccine is used to prevent not only varicella in children but also HZ in adults in Japan. However, the clinical trial prior to approval targeted healthy adults aged ≥50 years, and the safety profiles for patients with underlying illnesses have been limited.

Thus, the present study focused on adults aged ≥50 years with particular underlying illnesses (i.e., malignancy, diabetes mellitus, autoimmune diseases, and chronic renal disease), which were reported to be high-risk conditions for HZ, and compared the reactogenicity of freeze-dried live attenuated varicella-zoster vaccine with that in healthy adults aged ≥50 years.

## Methods

### Setting and study subjects

A prospective cohort study was conducted to compare the safety of live attenuated varicella-zoster vaccine between patients with underlying illnesses and healthy adults. Study subjects included patients with malignancy, diabetes mellitus, autoimmune diseases, or chronic renal disease attending the collaborating hospitals, SOUSEIKAI, in Japan. This study was run between November 3, 2016 and November 24, 2017. All patients were Japanese adults aged ≥50 years; were regarded as having a health condition compatible with participation by their physicians; and in the case of childbearing-aged women, those who had taken appropriate birth control for the preceding 1 month and those who consented to continue birth control for 2 months after vaccination. Exclusion criteria included receipt of transfusion or a γ-globulin preparation within the preceding 3 months, or a large amount of γ-globulin preparation (≥200 mg/kg) within the preceding 6 months; a history of anaphylaxis due to vaccine components; participation in other clinical trials within the preceding 4 months; lactating women or pregnant women, including those with suspected pregnancy at enrollment or those desiring pregnancy during the study period; or other condition making participation inappropriate.

Patients with malignancy included those with (a history of) malignant solid tumor such as colon cancer, lung cancer, gastric cancer, liver cancer, breast cancer, prostate cancer (males), cervical cancer (females), or with malignant lymphoma or acute lymphocytic leukemia, who were in the remission stage at the time of enrollment. Among them, the following patients were excluded: those who received immunosuppressive chemotherapy or radiation therapy within the preceding 6 months (or were planned to receive it within 28 days after vaccination); for patients with acute lymphocytic leukemia, those who had reached the remission stage within the preceding 3 months, those whose number of lymphocyte was less than 500/mm^3^, those with a negative result on the delayed skin hypersensitivity test, those who received chemotherapy for remission maintenance using medications other than 6-mercaptopurine within the preceding 1 week (or were planned for it within 28 days after vaccination); and for patients with malignant solid tumor, those whose tumor development could not be controlled by surgery or chemotherapy, those whose tumor development was under control but who received immunosuppressive chemotherapy or radiation therapy within the preceding 6 months (or were planned for it within 28 days after vaccination).

The inclusion criteria for diabetes mellitus patients were: patients diagnosed with diabetes mellitus; those without diabetic neuropathy, diabetic retinopathy, or diabetic nephropathy; those whose diabetes was not caused by the side effects of immunosuppressants (corticosteroid, tacrolimus, etc.); and those who did not receive cortical hormones, immunosuppressants, or antiplatelet therapy including aspirin.

Regarding autoimmune diseases, patients with rheumatoid arthritis, systemic lupus erythematosus, collagen diseases, ulcerative colitis, etc. were candidates for enrollment. Among them, patients who received cortical hormones, immunosuppressants, biologic agents, or JAK inhibitors within the preceding 6 months (or were planned to receive them within 28 days after the vaccination) were excluded.

Patients with chronic renal diseases were regarded as those with findings compatible with renal disease on urinalysis, imaging, laboratory, or pathological examination. For example, patients whose albuminuria (≥30 mg/gCr) or proteinuria (≥0.15 g/gCr) had continued for ≥3 months, or those with eGFR levels of 46–59 mL/min/1.73 m^2^ were included. Patients receiving cortical hormones or immunosuppressants were excluded.

For comparison, healthy adults aged ≥50 years were also enrolled. Those with mild underlying illnesses such as hypertension and dyslipidemia, if well-controlled, were allowed to participate.

### Sample size calculation

A total of 1500 subjects (300 patients and 1200 healthy adults) were needed for enrollment based on the following calculation. Based on the results of a domestic clinical trial involving 259 healthy adults aged ≥50 years, the proportion of any adverse events (AEs) after vaccination was 51%, and the most uncommon events were fatigue and rash (2% for each) [9]. Assuming that patients with underlying illnesses had a 3-fold higher risk for the most uncommon AEs than healthy adults, 1283 subjects (257 patients and 1026 healthy adults) were required to obtain 80% power (β = 0.20) for detecting significant differences with an α level of 0.05. When considering loss to follow-up (10%), a total of 1500 subjects were needed.

### Information collection

At the time of enrollment, the physicians were asked to complete a standardized case reporting form to collect the following information: demographic characteristics such as date of birth, age at vaccination, sex; a history of HZ and, if present, date of diagnosis; a history of varicella-zoster vaccination and, if present, date of vaccination; a history of any diseases; underlying illnesses (i.e., malignancy, diabetes mellitus, autoimmune diseases, renal disease) and name of medications; laboratory data (i.e., white blood cell counts and fractions within the preceding 6 months) if available; and HbA1c level and duration from diagnosis for patients with diabetes mellitus; and eGFR level, creatinine level, and dialysis treatment for patients with chronic renal diseases.

### Vaccination

All subjects received one subcutaneous injection of 0.5 mL of Live attenuated varicella virus vaccine BIKEN (Lot Nos. VZ184, 189, 200**)** manufactured by The Research Foundation for Microbial Diseases of Osaka University. To avoid confusion, this varicella virus vaccine is called varicella-zoster vaccine in this paper. Each vaccine was supplied as a single-dose vial containing live attenuated Oka varicella-zoster virus (29,000–58,000 PFU). No adjuvant was included in the vaccine.

### Safety assessment

All subjects were carefully observed for signs of any reactions for 30 min after vaccination at the hospitals. In addition, they maintained a daily log of body temperature, symptoms related to the injection-site (erythema, swelling, induration, pain, itching, warmth, and others), systemic symptoms (rash and others), any medications, and hospitalization during the 28 days after vaccination. Thereafter, they reported any symptoms until the next visit to the study clinic. If subjects experienced erythema, swelling, or induration at the injection site, they also reported the length of the major axis. Major axis length < 2 cm was regarded as mild, and an axis length > 5 cm was regarded as severe. For the other local symptoms (i.e., pain, itching, warmth, and others) and systemic symptoms, they selected the severity (i.e., mild, moderate, or severe). In general, mild symptoms were regarded as unnecessary to treat and did not interfere with daily activities, moderate symptoms needed treatment or interfered with daily activities, and severe symptoms needed hospitalization and interfered with daily activities. As for fever, a temperature < 38.0 °C was defined as mild fever, and a temperature ≥ 39.0 °C was defined as severe fever.

According to their daily logs, the physicians transferred the information to the case reporting forms and offered their opinions whether the symptoms were related to the vaccination. After this review, a MedDRA code was assigned to each AE.

### Statistical analysis

Key safety measures included proportions of subjects with any AEs, severe AEs (SAEs), and vaccine-related AEs such as injection-site AEs and systemic AEs. In the analysis, the frequencies and 95% confidence intervals (CIs) of AEs were calculated. Stratified analyses were performed to examine the effect of the following variables on the safety assessment: study population (patients and healthy adults); age at vaccination (50–59, 60–69, and ≥ 70 years); and sex. The χ^2^ test or Fisher’s exact test was used to compare the frequency of AEs and their severity among the above-mentioned stratified groups. Student’s *t-*test was also used as appropriate. Furthermore, to assess the risk of AEs among patients compared to healthy adults, logistic regression analyses were also performed with adjustment for age categories and sex, and the odds ratios (ORs) and 95% CIs were obtained. All tests were 2-sided. All analyses were performed using SAS, version 9.4 (SAS Institute).

## Results

### Study population

**Fig. 1:**
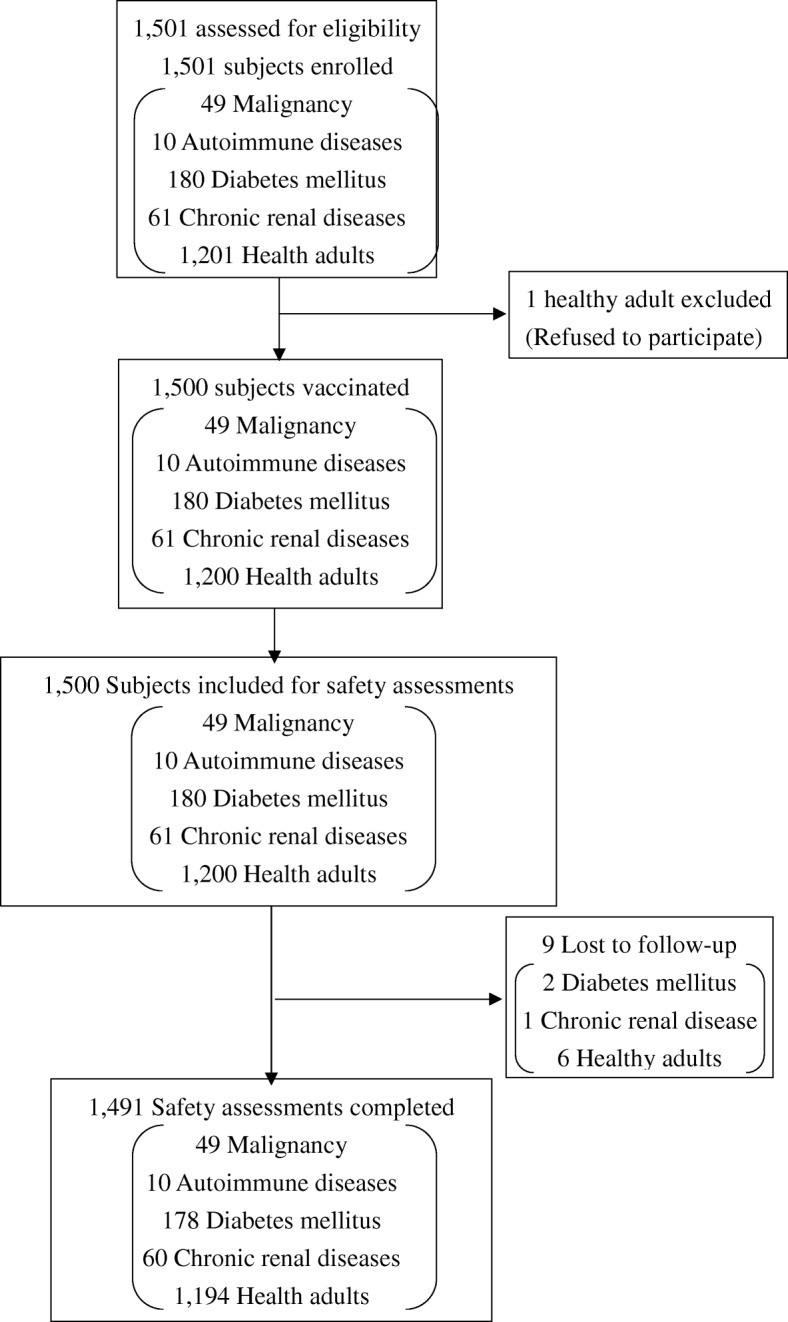
Flowchart of the study subjects

During the study period, 1201 healthy adults and 300 patients with underlying illnesses (49 malignancies, 180 diabetes mellitus, 10 autoimmune diseases, 61 renal diseases) were enrolled (Fig. 1). However, 1 healthy adult refused to participate after providing informed consent and was thus not vaccinated. Eventually, 1200 healthy adults and 300 patients with underlying illnesses were included in the safety analysis.

Table 1 shows the characteristics of the study subjects. Approximately half of the healthy adults were males, while male patients constituted more than half of the patients with other than autoimmune diseases. The mean age of healthy adults was 62.0 years, whereas older aged subjects were enrolled as patients with underlying illnesses, especially malignancy, diabetes mellitus, and chronic renal diseases. Patients with malignancy had a higher rate of HZ history and VZV vaccination history than healthy adults. Details of the sites of malignancy were: 11 breast cancer, 9 colon cancer, 8 prostate cancer, 7 gastric cancer, 3 uterine cancer, 3 lung cancer, 3 bladder cancer, 3 thyroid gland cancer, 1 gallbladder cancer, and 1 renal cancer. Regarding clinical information about diabetes mellitus, the HbA1c range was 5.0–11.0, and 41% of patients were considered to have well-controlled disease (i.e., HbA1c < 7.0%) at a mean of 8.0 years since diagnosis. Details of autoimmune diseases were: 6 Basedow’s disease, 3 autoimmune thyroiditis, 1 Sjögren’s syndrome with Basedow’s disease, and 1 Vogt-Koyanagi-Harada disease. Among patients with chronic renal diseases, ranges of creatinine levels and eGFR levels were 0.71–1.20 mg/dL and 46–59 mL/min/1.73 m^2^, respectively, suggesting that their disease activities were mild. None of the patients had undergone dialysis.

**Table 1:** Baseline characteristics of the study subjects

Characteristics		Healthy adults (*N* = 1200)	Patients with underlying illnesses (*N* = 300)	Patients with malignancy (*N* = 49)	Patients with diabetes mellitus (*N* = 180)	Patients with autoimmune diseases (*N* = 10)	Patients with chronic renal diseases (*N* = 61)
Sex	Male	607 (51%)	188 (63%)*	26 (53%)	120 (67%)*	2 (20%)**	40 (66%)*
Age (y)	Mean ± SD	62.0 ± 8.0	66.0 ± 8.0	71.0 ± 9.0*	65.0 ± 8.0*	61.0 ± 7.0	69.0 ± 8.0*
	50–59	530 (44%)	63 (21%)*	3 (6%)*	46 (26%)*	4 (40%)	10 (16%)*
	60–69	425 (35%)	129 (43%)	21 (43%)	80 (44%)	4 (40%)	24 (39%)
	70+	245 (20%)	108 (36%)	25 (51%)	54 (30%)	2 (20%)	27 (44%)
History of HZ	Present	155 (13%)	49 (16%)	11 (22%)**	28 (16%)	1 (10%)	9 (15%)
Previous vaccination	Present	3 (0.3%)	3 (1%)**	2 (4%)*	1 (1%)	0 (0%)	0 (0%)
White blood cells (/μL)	Mean ± SD	–	6289 ± 1685	5386 ± 1223	6505 ± 1811	6100 ± 520	5980 ± 1430
HbA1c (%)	Mean ± SD	–	–	–	7.0 ± 1.0	–	–
Duration of diabetes mellitus (y)	Mean ± SD	–	–	–	8.0 ± 7.0	–	–
Creatinine (mg/dL)	Mean ± SD	–	–	–	–	–	0.99 ± 0.15
eGFR (mL/min/1.73 m^2^)	Mean ± SD	–	–	–	–	–	53 ± 4
Dialysis	Present	–	–	–	–	–	0 (0%)

Data are expressed as n (%) unless otherwise indicated

*HZ* Herpes zoster, *SD* Standard deviation

*P < 0.05, **P < 0.1 (compared with the proportion of subjects among healthy adults)

### Safety assessment according to the study population

Table 2 shows the incidences of AEs within 28 days after vaccination. A total of 1623 events were reported from 603 healthy adults (50%), whereas 395 events were reported from 146 patients with underlying illnesses (49%). SAEs were reported from 2 healthy adults (fractures, acute cholecystitis) and 1 patient (disseminated mycobacteriosis), although both cases were considered to have no causal relationship with the vaccine. A total of 1362 events from 509 healthy adults and 328 events from 125 patients with underlying illnesses were diagnosed as vaccine-related AEs. The incidences of vaccine-related AEs were similar between healthy adults and patients with underlying illnesses (42% vs. 42%). Injection-site AEs were reported from 491 healthy adults (41%) and 118 patients (39%), and these were not significantly different between the groups. The incidences of systemic AEs were also similar between healthy adults (4%) and patients (4%). However, when each symptom was analyzed separately, the incidence of fever was slightly higher among patients with underlying illnesses (2%), more specifically malignancy patients and diabetes patients, compared with healthy adults (0.4%). When the risk of fever was examined among patients with underlying illnesses compared with healthy adults, a 4.1 times higher OR (95% CI: 1.2–14.1) was obtained. The age- and sex-adjusted OR reached the null value but remained 3.2 times higher (95% CI: 0.9–11.3) with marginal significance (*P* = 0.08).

**Table 2:** Adverse events within 28 days after vaccination in healthy adults and patients with underlying illnesses

Adverse events	Healthy adults (*N* = 1200)		Patients with underlying illnesses (*N* = 300)		Patients with malignancy (*N* = 49)		Patients with diabetes mellitus (*N* = 180)		Patients with autoimmune diseases (*N* = 10)		Patients with chronic renal diseases (*N* = 61)	
	No. of events	No. of subjects (%) (95% CI)	No. of events	No. of subjects (%) (95% CI)	No. of events	No. of subjects (%) (95% CI)	No. of events	No. of subjects (%) (95% CI)	No. of events	No. of subjects (%) (95% CI)	No. of events	No. of subjects (%) (95% CI)
Any AEs	1623	603 (50%) (47–53%)	395	146 (49%) (43–54%)	54	25 (51%) (36–66%)	234	85 (47%) (40–55%)	16	5 (50%) (19–81%)	91	31 (51%) (38–64%)
SAEs	2	2 (0.2%) (0–1%)	1	1 (0.3%) (0–2%)	0	0 (0%)	1	1 (0.6%) (0–3.0%)	0	0 (0%)	0	0 (0%)
Vaccine-related AEs	1362	509 (42%) (40–45%)	328	125 (42%) (36–47%)	44	20 (41%) (27–56%)	197	73 (41%) (33–48%)	14	5 (50%) (19–81%)	73	27 (44%) (32–58%)
Injection-site AEs	1306	491 (41%) (38–44%)	314	118 (39%) (34–45%)	41	18 (37%) (23–52%)	191	71 (39%) (32–47%)	12	4 (40%) (12–74%)	70	25 (41%) (29–54%)
Erythema	405	405 (34%) (31–37%)	94	94 (31%) (26–37%)	14	14 (29%) (17–43%)	52	52 (29%) (22–36%)	4	4 (40%) (12–74%)	24	24 (39%) (27–53%)
Itching	244	243 (20%) (18–23%)	60	59 (20%) (15–25%)	8	8 (16%) (7–30%)	39	38 (21%) (15–28%)	2	2 (20%) (3–56%)	11	11 (18%) (9–30%)
Swelling	179	179 (15%) (13–17%)	47	47 (16%) (12–20%)	9	9 (18%) (9–32%)	27	27 (15%) (10–21%)	1	1 (10%) (0.3–45%)	10	10 (16%) (8–28%)
Pain	183	182 (15%) (13–17%)	41	41 (14%) (10–18%)	3	3 (6%) (1–17%)**	28	28 (16%) (11–22%)	2	2 (20%) (3–56%)	8	8 (13%) (6–24%)
Warmth	170	170 (14%) (12–16%)	36	36 (12%) (9–16%)	5	5 (10%) (3–22%)	23	23 (13%) (8–19%)	2	2 (20%) (3–56%)	6	6 (10%) (4–20%)
Induration	124	124 (10%) (9–12%)	36	36 (12%) (9–16%)	2	2 (4%) (0.5–14%)	22	22 (12%) (8–18%)	1	1 (10%) (0.3–45%)	11	11 (18%) (9–30%)**
Eruption	1	1 (0.1%) (0–0.5%)	0	0 (0%)	0	0 (0%)	0	0 (0%)	0	0 (0%)	0	0 (0%)
Systemic AEs	56	46 (4%) (3–5%)	14	11 (4%) (2–6%)	3	2 (4%) (0.5–14%)	6	5 (3%) (0.9–6%)	2	1 (10%) (0.3–45%)	3	3 (5%) (1–14%)
Fever	6	5 (0.4%) (0.1–1.0%)	5	5 (2%) (0.5–4.0%)*	2	2 (4%) (0.5–14%)*	3	3 (2%) (0.3–5%)**	0	0 (0%)	0	0 (0%)
Headache	8	8 (0.7%) (0.3–1.0%)	2	2 (0.7%) (0.1–2.0%)	0	0 (0%)	0	0 (0%)	1	1 (10%) (0.3–45%)**	1	1 (2%) (0.04–9%)
Fatigue	5	5 (0.4%) (0.1–1.0%)	3	3 (1%) (0.2–3.0%)	1	1 (2%) (0.1–11%)	1	1 (0.6%) (0–3.0%)	1	1 (10%) (0.3–45%)*	0	0 (0%)
Rash	18	17 (1%) (0.8–2.0%)	1	1 (0.3%) (0.008–2.0%)	0	0 (0%)	1	1 (0.6%) (0–3.0%)	0	0 (0%)	0	0 (0%)
Others	19	17 (1%) (0.8–3%)	3	3 (1%) (0.2–3%)	0	0 (0%)	1	1 (0.6%) (0.01–3.0%)	0	0 (0%)	2	2 (3%) (0.4–11%)

*AE* Adverse event, *CI* Confidence interval, *SAE* Severe adverse event

*P < 0.05, **P < 0.1 (compared with the proportion of subjects among healthy adults)

Regarding the severity of AEs, no significant difference was observed in injection-site AEs between the groups. On the other hand, a higher incidence of mild to moderate fever was reported in patients with underlying illnesses (especially malignancy patients, diabetes patients) compared to healthy adults. In addition, mild headache and mild fatigue were found in one patient with autoimmune disease, and the incidences were higher than in healthy adults.

Most of the vaccine-related AEs occurred within 0–3 days (mean: 2 days, median: 2 days) after vaccination in both groups. Injection-site AEs were improved within 6 days, and systemic AEs were improved within a few days (data not shown).

### Safety assessment according to the history of herpes zoster

Table 3 shows the incidence of vaccine-related AEs according to the history of HZ. Among healthy adults, those with an HZ history were more likely to report injection-site AEs than those without an HZ history (53% vs 39%, *P* = 0.017). In particular, only erythema was significantly more common in those with an HZ history than in those without (43% vs. 32%, *P* = 0.026). The severity of erythema was mild to moderate, and it occurred most frequently the day after vaccination, with an average duration of 5 days (data not shown). When the risk of erythema was examined in healthy adults with an HZ history compared to those without, a 1.7 times higher OR (95%CI: 1.2–2.4) was observed even after adjustment for age and sex. Further, among patients with underlying illnesses, no significant differences were observed in the incidences of AEs between patients with and without a history of HZ (Table 3).

**Table 3:** Incidence of selected vaccine-related adverse events by a history of herpes zoster

Adverse events	Healthy adults		Patients with underlying illnesses		Patients with malignancy		Patients with diabetes mellitus		Patients with autoimmune diseases		Patients with chronic renal diseases	
	No. of events	No. of subjects (%) (95% CI)	No. of events	No. of subjects (%) (95% CI)	No. of events	No. of subjects (%) (95% CI)	No. of events	No. of subjects (%) (95% CI)	No. of events	No. of subjects (%) (95% CI)	No. of events	No. of subjects (%) (95% CI)
Subjects with HZ history	*N* = 155		*N* = 49		*N* = 11		*N* = 28		*N* = 1		*N* = 9	
Vaccine-related AEs	198	87 (56%) (48–64%)*	54	23 (47%) (33–62%)	8	4 (36%) (11–69%)	30	13 (46%) (28–66%)	6	1 (100%)	10	5 (56%) (21–86%)
Injection-site AEs	189	82 (53%) (45–61%)*	54	23 (47%) (33–62%)	8	4 (36%) (11–69%)	30	13 (46%) (28–66%)	6	1 (100%)	10	5 (56%) (21–86%)
Systemic AEs	9	8 (5%) (2–10%)	0	0 (0%)	0	0 (0%)	0	0 (0%)	0	0 (0%)	0	0 (0%)
Subjects without HZ history	*N* = 1045		*N* = 251		*N* = 38		*N* = 152		N = 9		*N* = 52	
Vaccine-related AEs	1164	422 (40%) (37–43%)	274	102 (41%) (35–47%)	36	16 (42%) (26–59%)	167	60 (39%) (32–48%)	8	4 (44%) (14–79%)	63	22 (42%) (29–57%)
Injection-site AEs	1117	409 (39%) (36–42%)	260	95 (38%) (32–44%)	33	14 (37%) (22–54%)	161	58 (38%) (30–46%)	6	3 (33%) (7–70%)	60	20 (38%) (25–53%)
Systemic AEs	47	38 (4%) (3–5%)	14	11 (4%) (2–8%)	3	2 (5%) (0.6–18%)	6	5 (3%) (1–8%)	2	1 (11%) (0.3–48%)	3	3 (6%) (1–16%)

*AE* Adverse event, *HZ* Herpes zoster

*P < 0.05, **P < 0.1 (compared with the proportion of reported subjects without HZ history within the category of subjects)

### Safety assessment in patients by disease severity

Additionally, the effect of disease condition on vaccine safety in patients with underlying illnesses was examined. In diabetes patients, no significant association was observed between the HbA1c level and the incidence of AEs (data not shown). However, those with a shorter time since diabetes diagnosis had a higher incidence of injection-site pain compared with those with a longer duration (within 4 years vs. 4–9 years vs. 10 years or more, 20% vs. 23% vs. 5%; *P* = 0.01). As for patients with renal diseases, those with a lower creatinine level had significantly higher rates of injection-site erythema (< 0.9 mg/dL vs. 0.9–1.08 mg/dL vs. > 1.08 mg/dL, 70% vs. 28% vs. 22%; *P* < 0.01), itching (40% vs. 11% vs. 4%; P < 0.01), pain (30% vs. 6% vs. 4%; P = 0.01), and induration (40% vs. 0% vs. 13%; *P* = 0.03), and those with a higher eGFR level had significantly higher rates of injection-site erythema (< 51 vs. 51–56 vs. more than 56 mL/min/1.73 m^2^, 29% vs. 32% vs. 55%; *P* = 0.04) and itching (12% vs. 5% vs. 36%; P = 0.03). No other significant differences were observed in AEs and background characteristics.

## Discussion

In the present study, no vaccine-related SAEs were observed in both patients with underlying illnesses and healthy adults. The incidence of AEs in healthy adults was almost the same as reported in domestic clinical trials for healthy adults > 50 years old (any AE: 50% vs. 56%; injection-site AEs: 41% vs. 50%) [10], suggesting that the present results are reliable. The present study also indicated that the incidences of most AEs were similar between healthy adults and patients with underlying illnesses, although the incidence of fever was significantly higher in patients than in healthy adults. In particular, fever was not observed in patients with autoimmune diseases and patients with chronic kidney disease, but only in patients with malignancy or diabetes. It is therefore considered that they are more likely to develop fever due to the background diseases rather than the vaccination itself. The period of emergence of fever in patients with underlying illnesses ranged from 0 to 5 days after vaccination, the extent of fever was mild to moderate, and all improved in 1–3 days, suggesting that it was a transient response. However, just as a precaution, patients with malignancy and diabetes patients should be aware of the possibility of fever for several days after vaccination.

Most of the previous studies that evaluated the safety of HZ vaccine were based on randomized, controlled trials. According to these studies, injection-site AEs were more commonly reported in the HZ-vaccinated group than in the placebo group, while the incidence of systemic AEs was similar between the HZ-vaccinated group and the placebo group, not only in elderly people with underlying illnesses, considered at high risk for HZ (AIDS, diabetes, steroid administration, autoimmune disease, renal disorder) [11–13], but also in healthy adults [14, 15]. These results suggested that the reported systemic AEs are less likely to be related to HZ vaccination. Furthermore, based on AE reports after ZOSTAVAX® had been used around the world for 10 years, injection-site AEs were the most frequently reported [16]. Therefore, this seemed to indicate that we need not be overly concerned about systemic AEs.

The present study also showed that healthy adults with an HZ history had a higher incidence of erythema after vaccination than those without. As far as we know, only one previous study examined vaccine safety by comparing 420 subjects with an HZ history and 13,254 subjects without an HZ history and showed that the incidence of SAEs during the 28 days after vaccination was similar between these groups (0.95% vs. 0.66%) [17]. However, the study targeted only the incidence of SAEs rather than all AEs or vaccine-related AEs, and, thus, the incidence of injection-site AEs including erythema was not reported. Since cellular immunity against VZV was activated by the HZ history [1], it is possible that the local reaction after vaccination was more likely to develop among those with a history of HZ. As additional information, however, erythema was self-controlled and recovered within an average of 5 days, and no severe erythema was observed in the present study.

Patients with diabetes are considered to have a high risk for HZ, since cellular immunity against VZV is lower than that of healthy adults [7, 18]. In the present study, the incidences of injection-site AEs and systemic AEs in diabetic patients were 39 and 3%, respectively, similar to healthy adults, irrespective of their HbA1c levels, although their disease condition, on the whole, tended to be mild. Further, patients with a shorter time since diabetes diagnosis had a higher incidence of injection-site pain. However, other AEs were similarly reported by patients, irrespective of time since diagnosis. Thus, a higher incidence of pain in patients with a shorter time since diagnosis may be obtained by chance. Therefore, we considered that the benefit of receiving a live attenuated varicella-zoster vaccine to prevent HZ and PHN exceeds the safety concerns, at least among such milder diabetes patients.

Patients with chronic renal diseases are also regarded as a high-risk group for HZ and would need vaccination. However, some injection-site AEs were reported more often from patients with lower creatinine levels or higher eGFR levels, with relatively mild disease. There is no possible explanation for why injection-site AEs were more frequently reported from milder renal disease patients. Further investigations of chronic renal disease patients may clarify the potential difference in AE occurrence after vaccination by disease severity.

As far as we know, few studies have compared the safety of a live attenuated HZ vaccine in patients with underlying illnesses with that in healthy adults, and only small-scale studies are available [19, 20]. In a study comparing safety in 10 diabetic patients and 10 healthy adults, no systemic AEs were observed in both groups [19]. In a study of 41 patients with rheumatism and 28 patients with osteoarthritis, 17 (25%) AEs occurred within 7 days after vaccination, of which 8 were injection-site AEs [20]. When investigating rare AEs after vaccination, it is difficult to detect AEs in such small-scale studies. From this point of view, the present study was a large-scale study comparing 1200 healthy adults and 300 patients with underlying illnesses, and the safety comparison is highly reliable. Although patients with diabetes and patients with chronic renal diseases in the present study had relatively mild disease, the evidence for the safety of these patients receiving a live attenuated HZ vaccine is valuable.

However, the present study has the following limitations. First, the number of patients with autoimmune diseases was too small and heterogeneous, which may not have been sufficient to examine disease-specific vaccine safety. In particular, statistical power may have been insufficient for systemic AEs, which usually occur with a low frequency, when comparing the incidence of disease-specific AEs. In addition, the cancer group was also heterogeneous, with small patient numbers per cancer type. As for vaccine safety in patients with autoimmune diseases, a previous study that included a larger number of patients with several kinds of autoimmune diseases did not identify any safety signal in the use of immunosuppressive therapies within 42 days after vaccination [21]. Second, the generalizability of the present study needs to be considered. Since the present vaccine contains a similar amount of live attenuated Oka virus as ZOSTAVAX®, the present results could be applicable to ZOSTAVAX® users. In the present study, however, diabetes patients accounted for 60% of patients with underlying illnesses, which means that the present patients may not be representative of the general population of patients with underlying illnesses. In addition, it is important to note that the present findings would be limited for malignancy patients in longer remission and without therapy for more than 6 months, non-severe diabetes (i.e., no organ damage) patients, autoimmune disease patients without immunosuppressive therapy, and patients with only mild renal disease. Third, since sex and age distributions differed between healthy adults and patients with underlying illnesses, the incidence of AEs may have been influenced by these background factors. In the present study, sex- and age-adjusted analyses were also performed, but the possibility of residual confounding cannot be excluded. Fourth, there was no primary outcome, since it was considered that comparing every AE outcome between healthy adults and patients with underlying illnesses was an important goal. However, it resulted in many comparisons in the analyses, which might run the risk of some spurious findings. In the present results, there was no possible explanation for why injection-site AEs were more common in those with milder renal diseases, which may be spurious.

To recommend vaccination for patients with underlying illnesses, evidence for vaccine efficacy is also needed. A retrospective cohort study of 463,541 patients aged 60 years or older with immune-mediated diseases reported that HZ vaccine was associated with a 39% (95%CI: 29–48%) decreased risk for HZ [21]. In a large-scale US study, the efficacy of HZ vaccine was 52% (95%CI: 44–61%) among subjects aged ≥65 years, and 63% (95%CI: 42–94%) among immunosuppressed patients [22]. In another study of 180,000 patients with chronic renal disease, vaccine efficacy was reported to be 51% (95%CI: 35–64%) among all patients and 54% (95%CI: 32–91%) among patients with diabetes mellitus [23]. Therefore, we believe that it is highly valuable to recommend vaccination for such patients with underlying illnesses, although it should be noted that live-attenuated varicella-zoster vaccine is contraindicated for some immunosuppressed patients (e.g. receiving cortical hormones, immunosuppressants including rituximab, chemotherapy, radiation, etc.).

## Conclusions

The present study confirmed the safety of freeze-dried live attenuated varicella-zoster vaccine even among patients with underlying illnesses who are at high risk for HZ. These results would be useful when providing vaccines to such patients.

## Abbreviations

AE: Adverse event
CI: Confidence interval
HZ: Herpes zoster
OR: Odds ratio
PHN: Post-herpetic neuralgia
SAE: Severe adverse event
SD: Standard deviation
VZV: Varicella-zoster virus
